# Examining Signatures of Natural Selection in Antifungal Resistance Genes Across *Aspergillus* Fungi

**DOI:** 10.3389/ffunb.2021.723051

**Published:** 2021-09-10

**Authors:** Renato Augusto Corrêa dos Santos, Matthew E. Mead, Jacob L. Steenwyk, Olga Rivero-Menéndez, Ana Alastruey-Izquierdo, Gustavo Henrique Goldman, Antonis Rokas

**Affiliations:** ^1^Departamento de Ciências Farmacêuticas, Faculdade de Ciências Farmacêuticas de Ribeirão Preto, Universidade de São Paulo, São Paulo, Brazil; ^2^Department of Biological Sciences, Vanderbilt University, Nashville, TN, United States; ^3^Medical Mycology Reference Laboratory, National Center for Microbiology, Instituto de Salud Carlos III, Madrid, Spain

**Keywords:** section *Fumigati*, antifungal drug resistance, positive selection, cryptic species, azole, *Aspergillus fumigatus*, echinocandin

## Abstract

Certain *Aspergillus* fungi cause aspergillosis, a set of diseases that typically affect immunocompromised individuals. Most cases of aspergillosis are caused by *Aspergillus fumigatus*, which infects millions of people annually. Some closely related so-called cryptic species, such as *Aspergillus lentulus*, can also cause aspergillosis, albeit at lower frequencies, and they are also clinically relevant. Few antifungal drugs are currently available for treating aspergillosis and there is increasing worldwide concern about the presence of antifungal drug resistance in *Aspergillus* species. Furthermore, isolates from both *A. fumigatus* and other *Aspergillus* pathogens exhibit substantial heterogeneity in their antifungal drug resistance profiles. To gain insights into the evolution of antifungal drug resistance genes in *Aspergillus*, we investigated signatures of positive selection in 41 genes known to be involved in drug resistance across 42 susceptible and resistant isolates from 12 *Aspergillus* section *Fumigati* species. Using codon-based site models of sequence evolution, we identified ten genes that contain 43 sites with signatures of ancient positive selection across our set of species. None of the sites that have experienced positive selection overlap with sites previously reported to be involved in drug resistance. These results identify sites that likely experienced ancient positive selection in *Aspergillus* genes involved in resistance to antifungal drugs and suggest that historical selective pressures on these genes likely differ from any current selective pressures imposed by antifungal drugs.

## Introduction

*Aspergillus fumigatus* is an important fungal pathogen that causes aspergillosis, a spectrum of diseases that includes aspergilloma, allergic bronchopulmonary aspergillosis, and invasive pulmonary aspergillosis (Latgé and Chamilos, 2019). A few other *Aspergillus* species, including closely related species that also belong to section *Fumigati*, are known to be pathogenic (Steenwyk et al., 2019; Rokas et al., 2020). Some of these pathogenic so-called cryptic species, such as *A. lentulus* (Balajee et al., 2005) and *A. fumigatiaffinis* (Hong et al., 2005), are morphologically similar and indistinguishable from each other and from *A. fumigatus* by classical clinical microbiology methods (Alastruey-Izquierdo et al., 2014).

The first-line drugs against aspergillosis are the azoles, with echinocandins or amphotericin B as alternative or complementary treatments (Denning and Bromley, 2015; Novak et al., 2020). Azoles such as itraconazole, voriconazole, and posaconazole are fungicidal to *Aspergillus* spp. and target the ergosterol biosynthesis pathway by inhibiting lanosterol 14α-demethylase (*cyp51A*), resulting in accumulation of 14-methylated sterols in the cell membrane. Altered membrane fluidity followed by disruption decreases the activity of membrane-bound enzymes, which in turn inhibits cell growth and proliferation (Pérez-Cantero et al., 2020). Echinocandins are fungistatic to *Aspergillus* spp. and act by inhibiting β-1,3-glucan synthase, encoded by *fks1* gene, the enzyme responsible for the synthesis of β-(1,3)-D-glucan, a major component of the fungal cell wall. Echinocandins disrupt the tips of hyphal walls, increasing the internal osmotic pressure and causing cell death (Nishiyama et al., 2005; Enoch et al., 2014).

Antifungal resistance is a worldwide concern, both in the clinic and in the field (Hagiwara et al., 2016; Verweij et al., 2016; Garcia-Rubio et al., 2017; Perlin et al., 2017; Fisher et al., 2018; Wassano et al., 2020). In *A. fumigatus*, resistance was first reported in 1997; since then, azole-resistant isolates have been reported in several different countries and are associated with therapy failure and increased mortality rates in immunocompromised patients (Wei et al., 2015). Additionally, closely related pathogenic species have been shown to differ from *A. fumigatus* in their drug susceptibility to amphotericin B and azoles (Alastruey-Izquierdo et al., 2014). For example, most *A. lentulus* isolates exhibit an increased resistance to several antifungal drugs (e.g., itraconazole, voriconazole, caspofungin, and amphotericin B) compared to *A. fumigatus* (Swilaiman et al., 2013).

Several molecular mechanisms are thought to be involved in antifungal drug resistance (Resendiz Sharpe et al., 2018; Chen et al., 2020b; Pérez-Cantero et al., 2020). Azole resistance mainly stems from mutations in the drug target gene, *cyp51A*, involving amino acid substitutions as well as tandem repeats (TRs) in its promoter region. In *A. fumigatus*, changes in the Cyp51A protein sequence that correlate with azole resistance include amino acid substitutions, such as in positions G54, G138, M220, and G448, or combinations of substitutions with TRs in the promoter region, such as TR34/L98H and TR46/Y121F/T289A (Wei et al., 2015; Beardsley et al., 2018). Changes in *cyp51A* expression have also been reported in several cases of drug-resistant *Aspergillus* species (Osherov et al., 2001). A mutation in *cyp51B*, a paralog of *cyp51A*, has also been linked to azole resistance in *A. fumigatus* (Gonzalez-Jimenez et al., 2020). In *Aspergillus flavus*, mutations in *cyp51C*, a third *cyp51* paralog, have also been associated with resistance (Sharma et al., 2018).

Several other non-*cyp51* genes have been associated with azole resistance (Chen et al., 2020b; Pérez-Cantero et al., 2020). For example, genes involved in transport, including the ATP-binding cassette (ABC) and the Major Facilitator Superfamily (MFS) (Slaven et al., 2002; Nascimento et al., 2003; da Silva Ferreira et al., 2004; Fraczek et al., 2013; Paul et al., 2013; Meneau et al., 2016; Chen et al., 2020a), ergosterol biosynthesis (e.g., *hmg1*, the HMG-CoA reductase enzyme that participates in the regulation of sterol synthesis in eukaryotes) (Rybak et al., 2019; Arai et al., 2021), stress response (e.g., calcium signaling pathway) (Chen et al., 2020a), mitochondrial processes (Wei et al., 2017; Li et al., 2020), and regulatory genes (Willger et al., 2008; Blosser and Cramer, 2012; Song et al., 2016, 2017; Hagiwara et al., 2017; Furukawa et al., 2020; Hortschansky et al., 2020) are implicated in azole resistance. Beyond azoles, echinocandin resistance is associated with mutations in the *fks1* gene in both *Candida* yeasts (Desnos-Ollivier et al., 2008; Garcia-Effron et al., 2008) and in *A. fumigatus* (Jiménez-Ortigosa et al., 2017; e Silva et al., 2020). Molecular mechanisms of resistance to amphotericin B have been hypothesized to be linked to mutations in genes involved in sterol biosynthesis that cause changes in the cell membrane composition, oxidative stress response, and cell wall (mainly the 1,3-α-glucan portion). However, these mechanisms are understudied among fungal pathogens from section *Fumigati* (Carolus et al., 2020).

Whether other pathogenic *Aspergillus* species differ from *A. fumigatus* in their resistance profiles to antifungals is a question of great interest (Alastruey-Izquierdo et al., 2014). Isolates of *A. fumigatus* are phenotypically heterogeneous (Fuller et al., 2016; Kowalski et al., 2016, 2019; Keller, 2017; Ries et al., 2019; Steenwyk et al., 2020a,b), and we recently showed that this heterogeneity extends to antifungal drug resistance among clinical isolates in *A. fumigatus* and closely related cryptic species, *A. lentulus* and *A. fumigatiaffinis* (Dos Santos et al., 2020b), needing examination of multiple isolates per species. Previous genomic examinations of azole resistance solely focused on *A. fumigatus* (Abdolrasouli et al., 2015; Garcia-Rubio et al., 2018). Recently, Parent-Michaud et al. (2020) explored mechanisms of antifungal resistance in the pathogenic species *A. thermomutatus* and *A. turcosus*, in particular *cyp51A* and efflux-pump-mediated drug resistance, analyzing orthologs of the ABC transporters previously associated with azole resistance, such as *cdr1B, AfuMDR1-4*, and *atrF*. Despite these advances, studies that examine the evolution of drug resistance genes across several species of section *Fumigati* are lacking.

To address this gap of knowledge, we examined signatures of ancient positive selection in genes known to be involved in drug resistance across 42 isolates from 12 *Aspergillus* species in section *Fumigati*. To account for heterogeneity in antifungal susceptibility among isolates of closely related species, we were particularly interested in verifying whether resistant isolates were available for all clades of section *Fumigati* sequenced so far, and in identifying sites that have experienced positive selection across isolates of different species. We identified resistant and susceptible isolates across all the *Fumigati* clades. Among sites under positive selection, none were previously reported to be associated with antifungal resistance, suggesting that the selective pressure imposed by drugs today is not the same as the ancient selective pressure in the same genes.

## Materials and Methods

### Data Collection and Ortholog Identification

We selected genomes of 42 isolates from 12 species in *Aspergillus* section *Fumigati* for analysis and used *Aspergillus clavatus* (section *Clavati*) as an outgroup (Supplementary Table 1). We followed a recent classification of *Aspergillus* (Houbraken et al., 2020) when referring to specific series or sections. Augustus 3.1.1 (Stanke et al., 2004) was used for gene prediction in cases of genomes that lacked a publicly available gene annotation. To identify gene orthogroups, we used DIAMOND (Buchfink et al., 2015) in an all-versus-all protein search of all proteomes, followed by OrthoFinder v.2.3.3 (Emms and Kelly, 2019) to group proteins into orthogroups.

### Antifungal Susceptibility Testing and Classification of Fungal Isolates

Antifungal susceptibility testing (AST) was conducted for four *Aspergillus* isolates and these results were merged with those from a previous publication (Dos Santos et al., 2020a). We used the EUCAST (European Committee for Antimicrobial Susceptibility Testing) reference microdilution method version 9.3.2 (https://www.eucast.org/fileadmin/src/media/PDFs/EUCAST_files/AFST/Files/EUCAST_E_Def_9.3.2_Mould_testing_definitive_revised_2020.pdf) (Arendrup et al., 2015), in which isolates are grown on plates with increasing drug concentrations and the first concentration in which growth is inhibited (MIC) is recorded. For the four isolates of *A. hiratsukae* and *A. felis* recently sequenced by our group (Dos Santos et al., 2020a), we tested their susceptibility to four antifungal drug classes as previously described (Dos Santos et al., 2020b).

For isolates and species that were not in our collection and did not undergo AST, we searched the literature for reports of drug resistance (Supplementary Table 2). Whenever it was not EUCAST, the classification that the paper used was taken. When the EUCAST method was employed, isolates were classified based on the breakpoint values established for *A. fumigatus* and according to the following guide: https://www.eucast.org/fileadmin/src/media/PDFs/EUCAST_files/AFST/Clinical_breakpoints/AFST_BP_v10.0_200204_updatd_links_200924.pdf.

### Identification of Gene Markers and Phylogenetic Inference

To reconstruct the phylogeny of our isolates in section *Fumigati* (including the outgroup species, *A. clavatus*), we retrieved the sequences of the beta-tubulin (*benA*), calmodulin (*CaM*), actin (*act*), and the RNA polymerase II second-largest subunit (*RPB2*) gene markers. Each individual gene was aligned with MAFFT v.7.397 option L-INS-i (Katoh and Standley, 2013), followed by generation of a supermatrix consisting of the individual gene partitions with FASconCAT (Kück and Meusemann, 2010). The supermatrix was used as input in IQ-TREE v.2.0.3 option ‘-m MFP+MERGE' (Minh et al., 2020), with model selection based on the greedy strategy in ModelFinder (Lanfear et al., 2012; Kalyaanamoorthy et al., 2017), and using 1,000 bootstrap replicates to assess bipartition support (IQ-TREE -b 1000 option). The interactive web tool iTOL (Letunic and Bork, 2019) was used for tree visualization.

### Selecting Genes Involved in Antifungal Drug Resistance

We analyzed all genes previously studied in the context of antifungal drug resistance, in particular those connected to the azole class of drugs, that were present in single-copy or fewer across all isolates in our orthogroup analyses (Supplementary Tables 3, 4). Genes where more than four isolates (10%) lacked an ortholog were excluded. To avoid inconsistencies in how proteins were annotated in each genome, the corresponding coding sequences for all proteins of interest were recovered from NCBI using NCBI efetch v.13.8 (options–format fasta_cds_na–db protein). All coding sequences were converted to amino acids using the transeq function in EMBOSS v.6.6.0.0 (Rice et al., 2000), and aligned with MAFFT v.7.397 option L-INS-i (Katoh and Standley, 2013). Nucleotide sequences were then threaded onto the protein alignment using pal2nal (Suyama et al., 2006). Alignments were visually inspected with Jalview v.2.10.3 (Waterhouse et al., 2009).

### Analyzing Sites for Evidence of Positive Selection

The evolutionary rate ratio of dN/dS (also known as ‘*ω*') assesses the rate of non-synonymous substitutions to the rate of synonymous substitutions in DNA codon sequence alignments. Values of *ω* > 1 are suggestive of positive selection, whereas *ω* = 1 and *ω* < 1 are suggestive of neutral evolution and negative selection, respectively. To determine dN and dS values, we used codeml from PAML v.4.9i (Yang, 2007) with the following parameters: runmode = 0, seqtype = 1, CodonFreq = 2, ndata = 1, clock = 0, model = 0, icode = 0, fix_omega = 0, omega =.4, and cleandata = 1. We used two “site models” of codon evolution, the M7 model (beta model) that considers one *ω* across all isolates, plus 10 site classes with *ω* ≤1, and the M8 model (beta model and *ω* model) that considers 11 classes, 10 with *ω* ≤ 1 and one additional class with *ω* > 1. We also tested models M1a (two classes: 0 ≤ *ω* <1 and *ω* = 1) and M2a (three classes: 0 ≤ *ω* < 1, *ω* = 1, and *ω* > 1). To test whether positive selection occurred in a given gene, the log-likelihood values (ln*L*) from the two models were used in a likelihood ratio test (LRT), with M7 being the null model and M8 the alternative model (the same test was applied to M1a vs. M2a). The LRT value for each gene was compared to a χ*2* value (Jeffares et al., 2015). For genes that rejected the null hypothesis, the Bayes Empirical Bayes (BEB) analysis (Yang et al., 2005) was used to detect positively selected sites.

**Figure 1:**
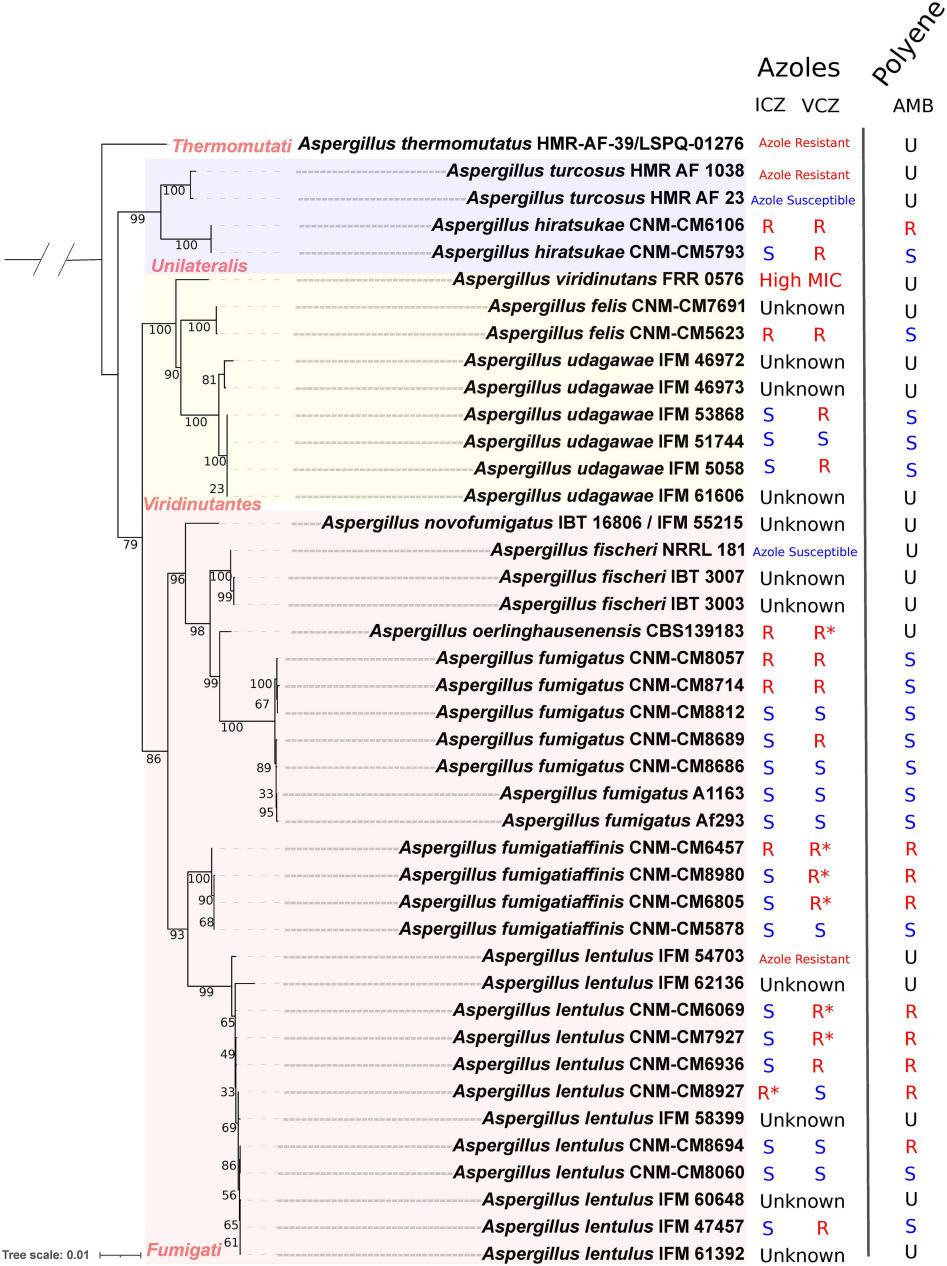
Phylogenetic reconstruction of strains in section *Fumigati* with sequenced genomes and the different susceptibility levels to different antifungals (the outgroup is not shown). We observed that the four sequenced Series (*Fumigati, Unilateralis, Viridinutantes, Thermomutati*) have members that exhibit resistance to at least one azole (marked in red in the internal nodes). ITC, itraconazole; VCZ, voriconazole; “R”, resistant strain; “S”, susceptible strain; “U”, AST (EUCAST) information is not available. “Azole resistant” or “Azole susceptible” means that this general pattern was reported in the literature. ^*^EUCAST has designated this an Area of Technical Uncertainty (ATU), that corresponds to an MIC value where the categorization is doubtful.

Given the extensive use of the *A. fumigatus* A1163 strain in laboratory studies, sequences in this organism were used as the reference to describe sites under positive selection in section *Fumigati*. A Python script was developed to recover the sites in the reference sequence (*checkPositionsAfterGaps*.py) for each site under positive selection. In cases where a gene was missing for one or more isolates, *treehouse* (Steenwyk and Rokas, 2019) was used to prune taxa from the species tree (Figure 1) to match the taxa in the gene tree. Given the importance of the alignment quality in evolutionary studies (Sackton, 2020), we employed GBlocks for codons with default parameter settings (Castresana, 2000) to identify poorly aligned regions.

## Results

### Isolates of Section *Fumigati* Species Exhibit Heterogeneity in Their Drug Resistance Profiles

To investigate the evolution of genes involved in antifungal resistance in members of *Aspergillus* section *Fumigati*, we studied the genomes of 42 isolates from 12 species from Europe (24), Asia (9), America (7), Oceania (1), and unknown (1). Most isolates correspond to clinical isolates, but some were environmental soil samples (Supplementary Tables 1, 2).

Using four phylogenetic gene markers (*benA, CaM, RPB2*, and *act*), we reconstructed the evolutionary history of these 42 section *Fumigati* isolates, using *A. clavatus* as the outgroup (Figure 1). Our phylogeny is consistent with previous studies (Houbraken et al., 2020; Dos Santos et al., 2020a,b) that assigned isolates of species into four series (or lineages within section *Fumigati*): *Fumigati* (*A. novofumigatus, A. fischeri, A. oerlinghausenensis, A. fumigatus, A. fumigatiaffinis*, and *A. lentulus*), *Unilateralis* (*A. turcosus* and *A. hiratsukae*), *Viridinutantes* (*A. udagawae, A. viridinutans*, and *A. felis*), and *Thermomutati* (*A. thermomutatus*). A high consistency in the phylogenetic reconstruction is important since it is required for our analyses of ancient selection.

To gain insights into the relationship between genome evolution and drug resistance, we searched the literature for antifungal susceptibility tests (ASTs) on these isolates (Supplementary Table 2; Figure 1). The most common AST uses the European Committee on Antimicrobial Susceptibility Testing (EUCAST) protocol, which we used to classify isolates as susceptible or resistant based on *A. fumigatus* breakpoints. However, for some isolates, the Clinical Laboratory Standards Institute (CLSI) and the YeastOne panel procedures were used for some of the isolates included (Tamiya et al., 2015; Lyskova et al., 2018). In these cases, we relied on the conclusions and criteria established by the original authors (Fedorova et al., 2008; Tamiya et al., 2015; Houbraken et al., 2016; Kusuya et al., 2016; Lyskova et al., 2018; Parent-Michaud et al., 2019a,b; Talbot et al., 2019; Dos Santos et al., 2020b) in order to describe these strains as susceptible or resistant.

Using these antifungal resistance data and the reconstructed phylogeny, we were able to identify members of all four series in section *Fumigati* that are resistant to azoles (Figure 1). We previously phenotyped several clinical isolates of *A. fumigatus, A. fumigatiaffinis*, and *A. lentulus*, and showed that most *A. fumigatus* were susceptible to both azoles and to amphotericin B (AMB). We also have previously noticed that *A. fumigatiaffinis* and *A. lentulus* are overall more resistant to AMB relative to *A. fumigatus* (Dos Santos et al., 2020b). Here, we expanded our analysis to other cryptic species of section *Fumigati*. In the series *Viridinutantes*, we obtained the AST for *A. felis* CNM-CM5623, which showed resistance to ICZ and VCZ, but was susceptible to AMB. We also phenotyped two *A. hiratsukae* (series *Unilateralis*) isolates that were recently sequenced by our group (Dos Santos et al., 2020a). Interestingly, the CNM-CM6106 isolate was resistant to azoles and to AMB, whereas CNM-CM5793 was susceptible to AMB and to some but not all azoles. In the same series, we identified isolates of *A. turcosus* with sequenced genomes that varied with respect to azole resistance. Finally, the only sequenced isolate of *A. thermomutatus* (series *Thermomutati*) is known to be azole resistant but its resistance to AMB is currently unknown. A summary of the drug resistance profiles, where known, of our isolates is shown in Figure 1 and the full data are shown in Supplementary Tables 1, 2.

### A Few Sites in Genes Involved in Resistance to Azoles and Echinocandins Have Signatures of Ancient Positive Selection

We next aimed to identify sites that experienced ancient positive selection and whether these sites overlap with known sites involved in antifungal resistance (Supplementary Tables 3, 4). To avoid complications associated with inferring evolutionary events across multiple paralogs (Jeffares et al., 2015), we only analyzed those genes associated with drug resistance that were present in single copy across either all or most of the 42 isolates from section *Fumigati* with available genomes. From the 41 genes in our initial list (Supplementary Table 4), 10 were removed because eight (AFUB_047000, AFUB_016810, AFUB_013880, AFUB_038670, AFUB_062080, AFUB_099400, AFUB_036760, AFUB_045980) had paralogs in at least one isolate and two (AFUB_092980, AFUB_078550) were absent from many isolates. Fourteen genes were single-copy in all studied isolates, and 17 were single-copy with orthologs missing in a few isolates resulting in 31 genes that were used in downstream analyses.

Among the 31 genes, we individually calculated the average value of *ω* (the evolutionary rate ratio of dN/dS, which assesses the rate of non-synonymous substitutions to the rate of synonymous substitutions) across sites (Supplementary Table 4). The mean of average *ω* values across genes was low (0.11; min: 0.06; max: 0.37). To identify genes with sites under positive selection, we used the log likelihood estimates (*lnL*) generated by different site models (M8 vs. M7) in likelihood ratio tests (LRT). We also calculated the LRT statistics for comparison of the simpler models M1a and M2a but found that most comparisons were not significant (Supplementary Table 4); given that the M7 and M8 models are more sensitive, we focused on the results from the M8 vs. M7 comparisons. Eleven of the 31 genes rejected the null hypothesis (> 9.21; 2 d.f. and 0.01 sign. level) (Table 1), suggesting these genes have undergone positive selection (*ω* > 1). Among the eleven genes, we identified a damage resistance protein, transporters, regulators, and metabolic enzymes that had all been previously reported in literature as involved in azole resistance. In addition, we found that the gene encoding a drug target for echinocandins, *fks1*, has sites under positive selection.

**Table 1:** Genes with signatures of positive selection (the Likelihood Ratio Test rejected the null hypothesis).

**Gene**	**Category**	**Antifungal class**	**Justification of selection**	**Reference(s)**
*abcA*	Transporter	Azole	Overproduction of *AbcA* in *A. fumigatus* yielded increased azole resistance; *S. cerevisiae* expressing *abcA* were more resistant to fluconazole than the PDR5-deleted background strain.	Esquivel et al., 2020
*abcF*	Transporter	Azole	*Saccharomyces cerevisiae* expressing *abcF* had the strongest efflux activities, had the broadest range of substrate specificity, and were more resistant to fluconazole.	Esquivel et al., 2020
*atmA*	Kinase	Azole	*atmA* and *atrA* mutant populations grown under sub-inhibitory drug concentration resulted in voriconazole resistance and discrete alterations in *cyp51A* and/or the *Cdr1B* efflux transporter; these kinases are likely involved in genetic stability	Dos Reis et al., 2018
*atrA*	Kinase	Azole	*atmA* and *atrA* mutant populations grown under sub-inhibitory drug concentration resulted in voriconazole resistance and discrete alterations in *cyp51A* and/or the *Cdr1B* efflux transporter; these kinases are likely involved in genetic stability	Dos Reis et al., 2018
*erg4B*	Reductase	Azole	*erg4A* and *erg4B* null mutant displays remarkable increased susceptibility to antifungal azoles (but not their isolated mutants)	Long et al., 2017
*fks1*	Drug target	Echinocandin	hot spot FKS1 mutation E671Q might be responsible for the reduced susceptibility	e Silva et al., 2020
*mdr3*	Transporter	Azole	Overexpression of Mdr3, Mdr4 or both drug efflux pump genes of *A. fumigatus* and/or selection of drug target site mutations are linked to high levels of itraconazole resistance	Nascimento et al., 2003
*mot1*	Transcriptional regulator/ modulator	Azole	Involved in regulation of the negative cofactor 2 complex, which is a key regulator of drug resistance in *A. fumigatus*	Furukawa et al., 2020
*ramA*	Enzyme	Azole	Loss of *ramA* led to a Cyp51A/B-independent increase in resistance to triazole antifungal drugs	Norton et al., 2017
*tpo3*	Transporter	Azole	*tpo3* and *dur3* played important roles in susceptibility to ITC via a potential mechanism (*tpo3* and *dur3* → polyamine homeostasis → ROS content → ITC susceptibility)	Chen et al., 2020a
*dapB*	Damage resistance protein	Azole	Overexpression of *dapB* and *dapC* causes dysfunction of *erg5* and *erg11*, resulting in abnormal accumulation of sterol intermediates and further accentuating the sensitivity of *ΔdapA* strains to azoles	Song et al., 2016

**Figure 2:**
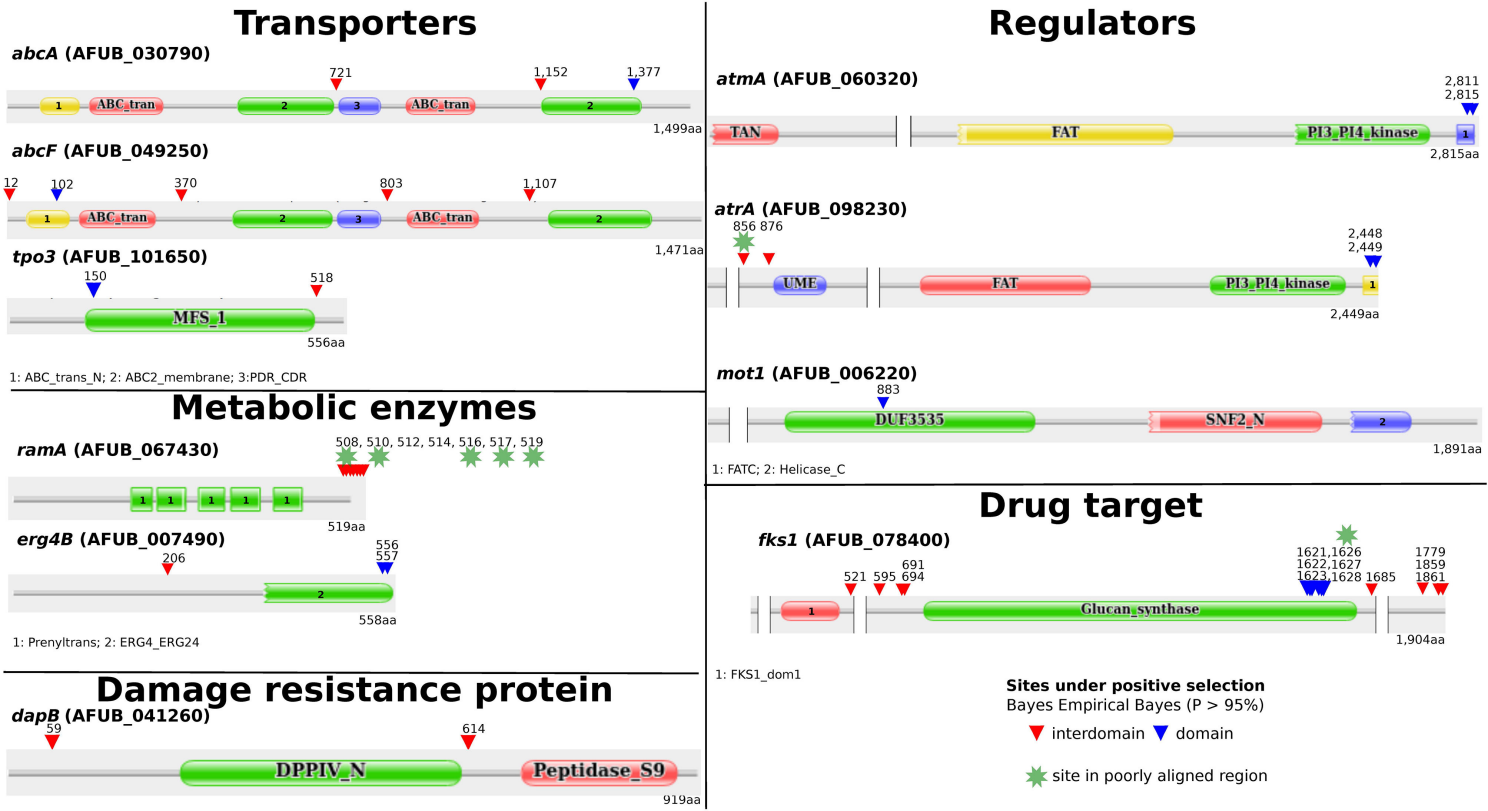
Protein annotation and annotated domains of genes for which the likelihood ratio tests (LRT) rejected the null hypothesis suggestive of positive selection among these genes. Among the eleven genes with significant LRT values, 10 presented sites under positive selection. Annotation of domains was carried out using the online Pfam database (http://pfam.xfam.org/). Proteins with long peptide stretches without any significant matches to Pfam domains were shortened (indicated by two bars with blank space in between). The gene products in *Aspergillus fumigatus* A1163 were used as reference.

Among the genes for which the LRT rejected the null hypothesis, ten out of eleven had sites under positive selection (*ω* > 1) according to the Bayes Empirical Bayes (BEB) analysis (> 0.95%) (Figure 2). In some genes (*fks1, ramA, erg4B, atmA*, and *atrA*), positively selected sites were clustered at the terminal region of their protein products, whereas in the damage resistance protein (*dapB*) and in transporter genes (*abcA, abcF*, and *tpo3*) these sites were distributed over their entire length. Finally, *mot1* only had one site under positive selection that was present in the DUF3535 domain of the protein.

To examine the impact of poor alignment quality on our inferences, we analyzed the location and alignment among sites under positive selection. Some of the sites clustered at terminal regions of some proteins (e.g., *ramA* and *erg4B*) appeared to be embedded in low quality alignment regions. Therefore, we employed GBlocks in the codon alignments to identify poorly aligned regions. In general, most alignment positions were maintained in all genes, with poor alignment regions including putative sites under positive selection identified in three genes: one position from *atrA* (856), one from *fks1* (1,626) and five clustered positions were eliminated from *ramA* (508, 510, 516, 517, and 519) (Figure 2; Supplementary Table 5). This resulted in 36 sites across ten genes with robust signatures of positive selection. The variation observed in sites under positive selection is consistent with our phylogeny (Figure 1). For example, we did not observe many differences between (closely related) strains of *A. fumigatus* but did observe differences between *A. fumigatus* and other (more distantly related) species.

We next searched for evidence in the literature of sites that we identified as potentially being positively selected in these ten genes as being involved in antifungal resistance susceptibility (Supplementary Table 5). To our knowledge, literature-based evidence of point mutations among our eleven genes is only available for *fks1* (Jiménez-Ortigosa et al., 2017; e Silva et al., 2020). Importantly, these sites are unchanged in all isolates with sequenced genomes included in our study. To our knowledge, none of the remaining ten genes with evidence of positive selection have been reported as having specific point mutations associated with antifungal susceptibility.

## Discussion

Here, we identified signatures of ancient positive selection in genes known for their involvement in antifungal resistance across sequenced strains of section *Fumigati*. Sites previously reported to contribute to antifungal resistance did not overlap with sites under positive selection, suggesting that any current selective pressures imposed by antifungals may differ from historical signatures of selection on these genes.

*Aspergillus fumigatus* is an important human pathogen (Latgé and Chamilos, 2019). Importantly, there has been an increased report of antifungal resistance across several isolates in *A. fumigatus* and cryptic species, in particular against azoles that comprise the main antifungal class used in clinical treatment. Azole resistance in *A. fumigatus* and closely related species is due to mechanisms in genes of different categories, including mutations and changes in expression of *cyp51* genes, transporters (ABC and MFS transporters), and genes involved in stress response, biofilm formation and mitochondria function (Pérez-Cantero et al., 2020).

The recent increase in availability of genomes in all groups of organisms has facilitated the analyses of adaptive evolution and the study of genes across whole genomes (Jeffares et al., 2015; Sackton, 2020). In fungi, the availability of genomes of *Aspergillus* has allowed the community to carry out species-wide comparative genomic analyses (de Vries et al., 2017; Mead et al., 2021). In order to study possible biological interactions shaping the evolution of species that might have favored antifungal resistance in *Aspergillus* section *Fumigati*, we used genomes of all sequenced species of this section to study positive selection with codon-based models as implemented in PAML (Yang, 2007). Importantly, we were able to measure or find in the literature tests of susceptibility across all clades of sequenced isolates in this section and classify them as resistant or susceptible to antifungals.

Several studies have classified cryptic species into resistant or susceptible categories based on their response to the main antifungal classes (Perlin et al., 2017; Imbert et al., 2020), although Imbert et al., emphasized that denser sampling of isolates and species would provide a more realistic and accurate classification. Interestingly, in all taxonomic series present in our study we identified azole resistance in at least one isolate. Unfortunately, several isolates with sequenced genomes have not been assayed in an AST (including isolates of *A. udagawae* and *A. novofumigatus*), and we therefore could not assign them to different susceptibility categories. Moreover, sequencing of additional members of section *Fumigati*, including unrepresented series *Brevipes, Spathulati, Neoglabri*, and *Fenneliarum* (Houbraken et al., 2020) would also increase the power of our analyses. Consistency of AST results across the different species was challenging, suggesting that future projects should rely on the same (e.g., EUCAST) or comparable AST methods.

We previously identified patterns of antifungal susceptibility among different isolates of pathogenic species in series *Fumigati* and suggested the potential for synergistic effects or trade-offs between different antifungals (Dos Santos et al., 2020b). Such patterns comprise important aspects to clinicians. Here, we showed that besides the clinical isolates phenotyped by our group (*A. fumigatus, A. fumigatiaffinis, A. lentulus, A. hiratsukae*, and *A. felis*) (Dos Santos et al., 2020a,b), there are no other reports of sequenced isolates that also have been tested for amphotericin B (AMB) using the EUCAST method. We previously observed an increased resistance to AMB in *A. fumigatiaffinis* relative to *A. fumigatus* (both in series *Fumigati*) (Dos Santos et al., 2020b), and these differences are highlighted in Figure 1. In the present study, however, we noticed that isolates susceptible to AMB were found among the recently sequenced species *A. hiratsukae* (series *Unilateralis*) and *A. felis* (series *Viridinutantes*) (Dos Santos et al., 2020a). Interestingly, in addition to isolates being resistant/susceptible to several drug classes, or being resistant to one class and not to others, we also observed cases of resistance to some azole antifungals but not to others, suggesting that isolates can develop a set of responses to drug that cannot be widely applied to all therapeutics.

Possible evolutionary mechanisms for the emergence of resistance in *A. fumigatus* in the clinic and the environment have been proposed (Buil et al., 2019; Schoustra et al., 2019). Schoustra et al. (2019) studied “hotspots,” environments that support growth, reproduction, and genetic variation of *A. fumigatus* and contain fungicides that facilitate emergence, amplification, and spread of resistance mutations. However, given the diversity of sources and the geographic distribution of strains across section *Fumigati*, in this study we were particularly interested in identifying sites that experienced ancient positive selection across the entire lineage instead of sites that may have more recently evolved to directly counteract current antifungals. To identify more ancient changes instead of the more recent ones studied by Schoustra et al. (2019), we employed site models, which allow the identification of sites that may have been subjected to repeated positive selection across the lineage (Sackton, 2020).

We identified several genes in the literature that have mutations previously linked to antifungal resistance (Supplementary Table 3). For example, several mutations in *cyp51A* are known for their involvement in antifungal resistance, such as G54, L98, G138, M220, G448, Y121, P216, F219, A284, Y431, G432, and G434 (Wei et al., 2015; Pérez-Cantero et al., 2020), as well as a mutation in *cyp51B* (G457S) (Gonzalez-Jimenez et al., 2020). Studying mitochondrial genes, (Li et al., 2020) identified sites important in antifungal resistance in the *cox10* gene, D234A and R243Q. A mutation associated with antifungal resistance (P88L) was also identified in the *hapE* gene, a conserved eukaryotic transcription factor (Hortschansky et al., 2020). Mutations (F262del, S305P, I412S, F390L) in the 3-hydroxy-3-methyl-glutaryl-coenzyme A (HMG-CoA) reductase-encoding gene, *hmg1*, have also been linked to antifungal resistance (Rybak et al., 2019; Gonzalez-Jimenez et al., 2020). However, none of these mutations were under positive selection in our results.

As stated previously, several studies have reported genes involved in antifungal resistance and susceptibility with a focus on specific mutations, the impact of knockouts, or overexpression of different genes. Previous works analyzed evolution of drug resistance in single species (Li et al., 2017). Recently, the evolution of antifungal resistance was studied in *Aspergillus fumigatus* (Rhodes et al., 2021). However, no previous work exploited the signatures of evolution in genes involved in antifungal resistance across all sequenced species in section *Fumigati*. We identified sites under positive selection in different regions of genes involved in azole resistance, including transporters, regulators, metabolic enzymes, and in *fks1*, the gene encoding the target of echinocandins. Interestingly, these sites were present in regions inside and outside conserved domains (Figure 1). For example, in the *abcA* (ABC transporter) gene, a site was found in the transmembrane domain (TMD) of the product. Mutations in ABC transporters are known to affect efflux and change the selectivity and susceptibility to substrates, including antifungals, in *Candida albicans* and *Saccharomyces cerevisiae* (Moreno et al., 2019). On the other hand, in the MFS *tpo3* transporter, previous experiments identified its role in importing polyamines that in turn seem to protect the cell from drug action in *A. fumigatus* (Chen et al., 2020a). Another example of a gene with sites under positive selection was *atmA*, a kinase known for involvement in DNA damage response, for which experiments performed with deletions combined with *atrA* (also with sites under positive selection) showed that null mutant *A. fumigatus* isolates had defective DNA repair and were azole-resistant (Dos Reis et al., 2018). Interestingly, sites identified in *atmA* were in the FATC domain, in which mutations are known to hamper the kinase activity (Awasthi et al., 2015).

Variation in these sites might have impacted how different *Aspergillus* species responded to selective pressures, which probably differs from the pressure imposed by antifungals today. Although sites under positive selection from this work are located in genes currently associated with antifungal resistance in *Aspergillus fumigatus*, it is indeed possible that they can be involved in increased survival of fungi in environments with selection pressures unrelated to those imposed by azole antifungals. Also of relevance was the fact that *cyp51A*, which is often associated with azole resistance, did not contain sites under positive selection while *fks1*, which is associated with echinocandin resistance, does contain sites that experienced ancient positive selection. Interestingly, unlike azoles (for which natural analogs are not known), echinocandin has structural analogs of natural origin (Denning, 2003) present in the environmental niches. Thus, the signatures of ancient positive selection observed in the *fks1* gene could reflect complex antagonistic interactions between *Aspergillus* fungi and their microbial competitors.

Further work will be able to address whether sites under positive selection in the studied genes confer advantages in survival of isolates exposed to azoles or echinocandins. Moreover, given the heterogeneity observed across different *Aspergillus* strains (Dos Santos et al., 2020a), population genetic analyses with respect to evolution of drug resistance in species with various sequenced genomes (e.g., *A. fumigatus*) is also an important next step.

## Data Availability Statement

Publicly available datasets were analyzed in this study. This data can be found here: All genomes included in this study are available from the NCBI GenBank database. Assembly accession numbers are presented in Supplementary Table 1, including the reference when a link is available. The data and scripts used in this project are available on the Gitlab repository under https://gitlab.com/SantosRAC/Santosetal2021_evolutionGenesAntifungalsFumigati.

## Author Contributions

RS, JS, MM, AA-I, GHG, and AR designed the experiments. OR-M performed the experiments. RS ran bioinformatic analyses. RS, MM, JS, and AR wrote the manuscript. All authors revised the manuscript.

## Funding

RS was supported by the Brazilian São Paulo Research Foundation (FAPESP) grant numbers 2017/21983-3 and 2019/07526-4. JS and AR are supported by the Howard Hughes Medical Institute through the James H. Gilliam Fellowships for Advanced Study Program. AR's laboratory received additional support from a Discovery grant from Vanderbilt University, the Burroughs Wellcome Fund, the National Science Foundation (DEB-1442113), and the National Institutes of Health/National Institute of Allergy and Infectious Diseases (R56AI146096). GHG was supported by FAPESP (2016/07870-9) and Conselho Nacional de Desenvolvimento Cientifico e Tecnologico (CNPq).

## Conflict of Interest

AR is a scientific consultant for LifeMine Therapeutics, Inc. The remaining authors declare that the research was conducted in the absence of any commercial or financial relationships that could be construed as a potential conflict of interest.

## Publisher's Note

All claims expressed in this article are solely those of the authors and do not necessarily represent those of their affiliated organizations, or those of the publisher, the editors and the reviewers. Any product that may be evaluated in this article, or claim that may be made by its manufacturer, is not guaranteed or endorsed by the publisher.
